# The impact of polymer coatings on magnetite nanoparticles performance as MRI contrast agents: a comparative study

**DOI:** 10.1186/s40199-015-0124-7

**Published:** 2015-09-17

**Authors:** Maryam Khalkhali, Kobra Rostamizadeh, Somayeh Sadighian, Farhad Khoeini, Mehran Naghibi, Mehrdad Hamidi

**Affiliations:** Department of Physics, Faculty of Science, University of Zanjan, Zanjan, Iran; Zanjan Pharmaceutical Nanotechnology Research Center, Zanjan University of Medical Sciences, Zanjan, Iran; Department of Medicinal Chemistry, School of Pharmacy, Zanjan University of Medical Sciences, Postal Code 45139-56184 Zanjan, Iran; Department of Pharmaceutical Biomaterials, School of Pharmacy, Zanjan University of Medical Sciences, Zanjan, Iran; Shahid Beheshti University of Medical Sciences, Tehran, Iran

## Abstract

**Background:**

Superparamagnetic iron oxide nanoparticles (SPIONs) are the most commonly used negative MRI contrast agent which affect the transverse (T_2_) relaxation time. The aim of the present study was to investigate the impact of various polymeric coatings on the performance of magnetite nanoparticles as MRI contrast agents.

**Methods:**

Ferrofluids based on magnetite (Fe_3_O_4_) nanoparticles (SPIONs) were synthesized via chemical co-precipitation method and coated with different biocompatible polymer coatings including mPEG-PCL, chitosan and dextran.

**Results:**

The bonding status of different polymers on the surface of the magnetite nanoparticles was confirmed by the Fourier transform infrared spectroscopy (FT-IR) and thermogravimetric analysis (TGA). The vibrating sample magnetometer (VSM) analysis confirmed the superparamagnetic behavior of all synthesized nanoparticles. The field–emission scanning electron microscopy (FE-SEM) indicated the formation of quasi-spherical nanostructures with the final average particle size of 12–55 nm depending on the type of polymer coating, and X-ray diffraction (XRD) determined inverse spinel structure of magnetite nanoparticles. The ferrofluids demonstrated sufficient colloidal stability in deionized water with the zeta potentials of −24.2, −16.9, +31.6 and −21 mV for the naked SPIONs, and for dextran, chitosan and mPEG-PCL coated SPIONs, respectively. Finally, the magnetic relaxivities of water based ferrofluids were measured on a 1.5T clinical MRI instrument. The r_2_/r_1_ value was calculated to be 17.21, 19.42 and 20.71 for the dextran, chitosan and mPEG-PCL coated SPIONs, respectively.

**Conclusions:**

The findings demonstrated that the value of r_2_/r_1_ ratio of mPEG-PCL modified SPIONs is higher than that of some commercial contrast agents. Therefore, it can be considered as a promising candidate for T_2_ MRI contrast agent.

## Introduction

Magnetic resonance imaging (MRI) is one of the noninvasive powerful imaging techniques with very high spatial resolution that allows precise determination of the 3D shape for differentiate soft body tissue. In order to make an accurate diagnosis and improve the intrinsic contrast between normal tissues and lesions, there is a need to use exogenous contrast agents. Contrast agents in clinic are classified into two categories [1]. The most commonly used MRI contrast agents are those that reduce the longitudinal (T_1_) relaxation time and cause positive contrast enhancement based on the paramagnetic ions including chelate complexes of gadolinium (Gd^3+^) or manganese (Mn^2+^). Due to some toxicity issues related to gadolinium [2], nowadays, there is a growing interest in negative contrast agents based on magnetic iron oxide nanoparticles (SPIONs) affect the transverse (T_2_) relaxation time and cause darker state in the T_2_-weighted image wherever accumulate in tissue [3]. As compared to gadolinium compounds, superparamagnetic iron oxide nanoparticles show the advantages of tunable size and shape, as well as possibility of surface modification and more effectiveness at lower concentrations because of their superparamagnetic property [4]. The effectiveness of SPIONs can be limited by their high surface area to volume ratio which leads to an increase in surface energy and tendency to agglomeration. This phenomena consequently makes them recognized by the macrophage system and reduce their circulation time. To overcome this shortcoming, one approach is to modify nanoparticles surface with various surface stabilizing agents that ensure their stability, biodegradability, non-toxicity as well as prolonging their circulation time in vivo. Although surface modification is successful in prolonging the SPIONs circulation time in vivo, according to the Koening – Kellar model, they could also influence the longitudinal (r_1_) and transverse (r_2_) relaxivities characteristics of SPIONs as a result of change in size, composition, accumulation situation in the biological environment, magnetization, hydrophilicity and surface properties [5–7].

Recently, Xie et al. [8] have prepared superparamagnetic iron oxide nanoparticles (SPIONs) coated with polyethylene glycol (PEG), PEG/PEI (poly ethyleneimine) and PEG/PEI/Tween 80 by the thermal decomposition of Fe(acac)_3_ and investigated their in vivo MRI contrast effects in the mouse brains. The results showed different vascular imaging effects after 24 h intravenous injection of the synthesized ferrofluids. Ma et al. [9] explored SPION-based MRI contrast agents by a polyol method. SPIONs entrapped into albumin nanospheres and then folic acid as targeting agent was conjugated onto the surface of nanoparticles. The r_2_/r_1_ value of resultant ferrofluids was around 40 indicating a strong T_2_ shortening effect. Ahmad et al. [10] reported synthesis of chitosan-coated nickel-ferrite (NiFe_2_O_4_) nanoparticles by a chemical coprecipitation method. The coated nanoparticles were cylindrical in shape and were studied as both T_1_ and T_2_ contrast agents in MRI. The T_1_ and T_2_ relaxivities were 0.858 ± 0.04 and 1.71 ± 0.03 mM^−1^ s^−1^, respectively. In animal study, both a 25 % signal enhancement in the T_1_-weighted image and a 71 % signal loss in the T_2_-weighted image were observed. This result demonstrated chitosan-coated nickel-ferrite nanoparticles potential as both T_1_ and T_2_ contrast agents in MRI.

According to the literature [5], it is clear that by careful selection of different coatings on SPIONs, it is possible to provide significant improvement in magnetic resonance activity. The aim of this contribution was to prepare the ferrofluids based on Fe_3_O_4_ magnetic nanoparticles (SPIONs) stabilized with various biocompatible polymer coatings such as dextran, chitosan and mPEG-PCL in order to elucidate the influence of the polymer type on the corresponding longitudinal (r_1_) and transverse (r_2_) relaxivities. SPIONs were characterized by the Fourier transform infrared spectroscopy (FT-IR), Dynamic Light Scattering (DLS) technique, field–emission scanning electron microscopy (FE-SEM), vibrating sample magnetometer (VSM) analysis, and X-ray diffraction (XRD). Finally T_1_ and T_2_ weighted phantom MRI images were obtained at a series of colloidal suspension of nanoparticles with different iron concentrations using 1.5 T MRI.

## Experimental

### Materials and method

Ferrous chloride tetrahydrate (FeCl_2_ · 4H_2_O), ferric chloride hexahydrate (FeCl_3_.6H_2_O), ammonium hydroxide, acetic acid, dextran (M_w_ ≈ 13–23 kDa), poly vinylalcohol (M_w_ ≈ 13–23 kDa) and dichloromethane all were purchased from Merck (Germany). Chitosan of molecular weight in the range of 10^5^–3 × 10^5^ g/mol and degree of deacetylation ≥ 75 %, poly (ethylene glycol) monomethyl ether (mPEG, 5000 g/mol), ε-caprolactone and stannous octoate were purchased from Sigma. Ethanol (96 %) and oleic acid were provided by Kimia alcohol (Iran) and Fluka (Switzerland), respectively. All chemicals used as received without further purification. mPEG-PCL copolymer with the average molecular weight of 13 kDa was synthesized and characterized. The detailed procedures of the synthesis of mPEG-PCL copolymer and its corresponding characterization have been described in our previous paper [11].

### Synthesis of naked superparamagnetic iron oxide nanoparticles (SPIONs)

Naked magnetite nanoparticles (SPIONs) were synthesized via alkaline coprecipitation of Fe^2+^ and Fe^3+^ ions in aqueous solution [12]. Briefly, a mixture of iron (II) chloride and iron (III) chloride (1:2, molar ratio) dissolved in 45 mL deionized water and put into a three-neck flask and mechanically stirred at 80 °C. Then, 4 mL NH_4_OH (25 wt %) was added dropwise to the solution under nitrogen protection and the mixture was continuously stirred for another 30 min to complete the reaction. The resultant SPIONs were collected by a 1.4 T magnet, and washed several times with ethanol and deionized water to eliminate excess ammonia and finally dried at 60 °C under vacuum for one day. The yield of reaction was 80 %.

### Synthesis of chitosan coated magnetite nanoparticles

Chitosan coated magnetite nanoparticles were synthesized according to the previous published method [13]. Briefly, 0.2 g of the naked magnetite nanoparticles prepared in the previous step were dispersed in 0.5 % chitosan solution (0.5 g chitosan dissolved in 100 mL acetic acid buffer with pH = 4.8) using an ultrasonic bath for 30 min at 60 °C and the mixture stirred mechanically at room temperature for 12 h and a black homogeneous suspension was obtained. During this process surface of nanoparticles were coated by chitosan and the resulting black precipitate was separated by a permanent magnet and washed five times with deionized water and dried at vacuum conditions.

### Synthesis of dextran coated magnetite nanoparticles

Synthesis of dextran coated SPIONs were adapted from the literature [14]. In a typical procedure, FeCl_3_ · 6H_2_O (12 mmol), FeCl_2_ · 4H_2_O (6 mmol), and 1.45 g dextran were dissolved in 150 mL deionized water. The mixture was ultrasonicated for 10 min at room temperature whilst pure nitrogen was bubbled into, then, followed by the addition of 4 M potassium hydroxide. After ultrasonication of mixture at 60 °C under nitrogen atmosphere for 60 min, the dark suspension was obtained. The black product was separated by centrifugation for 10 min at 14000 rpm and washed five times with absolute ethanol and deionized water. The final product was dried at room temperature.

### Synthesis of mPEG-PCL coated SPIONs (Magnetic micelles)

The synthesis followed the procedure performed by Meerod et al. [15]. In essence, a mixture of iron(II) chloride and iron(III) chloride (1:2, molar ratio) were dissolved in 45 mL deionized water. Then, 4 mL aqueous ammonia (25 %) and 250 μL oleic acid was added to the solution and stirred for 30 min under the N_2_ flow. The dark precipitant was isolated by a magnet and thoroughly were washed with ethanol to remove excess oleic acid and dried at 60 °C under vacuum for 24 h. Afterwards, 10 mg of mPEG-PCL and 2 mg of the oleic acid coated magnetite nanoparticles were dispersed in 2 ml dichloromethane, then the mixture was emulsified in 10 mL of 0.5 % (w/v) PVA aqueous solution. Dichloromethane was evaporated slowly by stirring overnight at room temperature and the magnetic micelles were formed.

### Characterization of nanoparticles

Fourier transform infrared (FT-IR) spectra for pure polymers and the naked and coated magnetite nanoparticles were recorded using Matson1000 FT-IR spectrometer (Unican, United States) with KBr pellets in the range of 400–4000 cm^−1^. Crystal structure and the phase analysis of SPIONs were studied by Bruker D8 X-ray diffractometer (Germany) with Cu *K*_*α*_ radiation (*λ* = 0.1540 nm) and diffraction patterns were collected in the diffraction angle in the range of 2θ = 5-70° at an accelerating voltage of 40 kV. PANalytical X’pert high score software was used for data analysis. Hydrodynamic diameter, zeta-potentials of nanoparticles and time dependent colloidal stability were characterized by dynamic light scattering (DLS) system (Zetasizer Nano ZEN 3600, Malvern Instruments Ltd., Worcestershire, United Kingdom)) at 25 °C. Thermogravimetric analysis of the dried samples were performed by a NETZSCH STA 409 PC/PG (Selb, Germany) at a heating rate of 10 K/min from 20 to 800 °C to monitor the mass loss of a known amount of polymer coated SPIONs. Vibrating sample magnetometer (VSM) (Lake shore 7400, United States) was employed to study the hysteresis loops and the magnetic properties of the magnetite nanoparticles at room temperature from −20000 to 20000 Oe. The iron concentration was measured by inductively coupled plasma atomic emission spectrometer (ICP) Optima 7300DV (United States). The particle size, structure and morphology of the naked and polymer coated magnetite nanoparticles were investigated by the field-emission scanning electron microscopy (FE-SEM) Mira 3-XMU (Tescan, United States).

### *In vitro* MRI studies (Relaxometry properties of the ferrofluids)

To assess the longitudinal (R_1_) and transverse (R_2_) relaxation rates, clinical 1.5 T whole body magnetic resonance (MR) scanner (Siemens Healthcare Avanto Germany) was used. T_1_ and T_2_ weighted phantom MRI images were obtained at a series of colloidal suspension of nanoparticles with iron concentrations of 0, 25, 50, 75, 100 and 200 μM. A number of spin echo sequence with repetition times (TR) of 1600 ms and varying echo time (TE) of 10, 43, 75, 108 and 140 ms (slice thickness: 7.5 mm, field of view (FOV): 238, Turbo factor: 18, matrix: 176 × 384) was used for getting T_2_ weighted images. The T_1_ weighted images were obtained at various repetition times of 100, 1550, 3150, 4750 and 6400 ms with an echo time of 18 ms, slice thickness:7.5 mm, field of view (FOV): 230, and matrix: 200 × 256 [16, 17].

Signal intensity of the spin echo sequence related to TE and TR is defined as [18]:

1 $$ I={M}_0\left[1- \exp \left(-\frac{TR}{T_1}\right)\right] $$

2 $$ I={M}_0 \exp \left(-\frac{TE}{T_2}\right) $$

Where *I* is the signal intensity which was measured with the help of DicomWorks 1.3.5 software within a manually drawn region of interest (ROI) for each sample. Relaxation rate R_1_ (1/T_1_) and R_2_ (1/T_2_) were calculated by using the eqs. 1 and 2 via mono-exponential curve fitting of the signal intensity vs. time (TE or TR). By plotting R_1_ and R_2_ over Fe concentration of synthesized ferrofluids, the slope indicates the specific relaxivity, r_1_ and r_2,_ respectively.

## Results and discussion

**Fig. 1:**
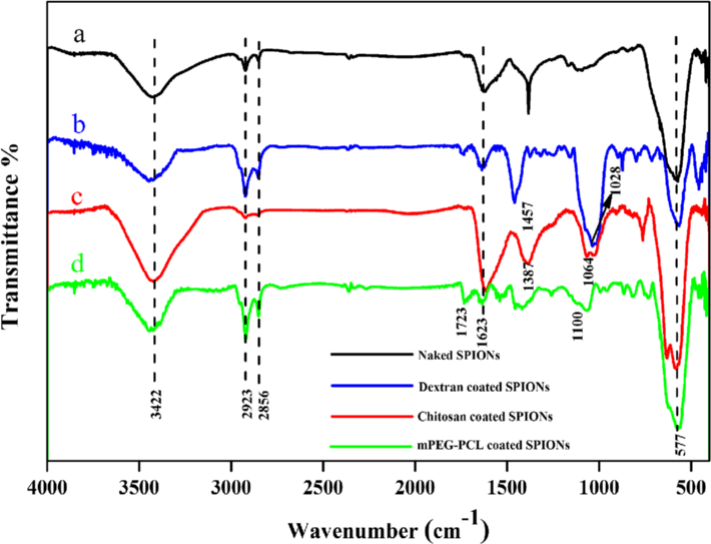
FT-IR spectra of **a** naked SPIONs, **b** dextran coated SPIONs, **c** chitosan coated SPIONs, **d** mPEG-PCL coated SPIONs

FT-IR spectral analysis was applied to confirm the introduction of different coatings on SPIONs. Figure 1 shows the characteristic peaks of the naked and polymer coated SPIONs. For all the samples, a main band at 577 cm^−1^ is attributed to the vibration of Fe-O [19]. For the FT-IR spectrum of the naked SPIONs (Fig. 1a), the absorption peak at 3422 cm^−1^ corresponds to stretching vibration of OH indicating the presence of the large number of hydroxyl groups on the surface of iron oxide particles which increase the agglomeration tendency of the synthesized SPIONs [20]. For the FT-IR spectrum of the dextran coated SPIONs, the absorption line at 1028 cm^−1^ is due to the absorption by the vibrational motion of the etheric bond (−C-O-), the signal at 3422 cm^−1^ is assignable to stretching vibration of the alcoholic hydroxyl (−OH), the peak centered at 1457 cm^−1^ is due to the bending vibration of C-H bond and the peak appeared at 2923 cm^−1^ is referred to the stretching vibration of -CH_2_- groups (Fig. 1b) [21]. FT-IR spectrum of the chitosan coated SPIONs is shown in Fig. 1c. The characteristic absorption peak at 1064 cm^−1^ can be attributed to the absorption by the vibrational motion of the C-O bond. The peak at 1387 cm^−1^ is due to the vibration of the CH_2_ group. The characteristic absorption peak at 1623 cm^−1^ can be referred to the N-H bending vibration of primary amine (NH_2_) and corresponding high intensity and broad peak of absorption can be explained by the fact that the hydrogen of primary amino group in chitosan form strong hydrogen bonding with the oxygen of magnetite [20]. Apparently, the above observations imply successful attachment of chitosan onto the surface of SPIONs. It can be seen that for mPEG-PCL coated SPIONs (Fig. 1d), the weak absorption at 1100 cm^−1^ and the small shoulder band at 1723 cm^−1^ is assignable to the C-O stretching of mPEG and carbonyl stretching of ester linkages of PCL which subsequently can be considered as an evidence for the copolymer attachment to the particle surface [15].

**Fig. 2:**
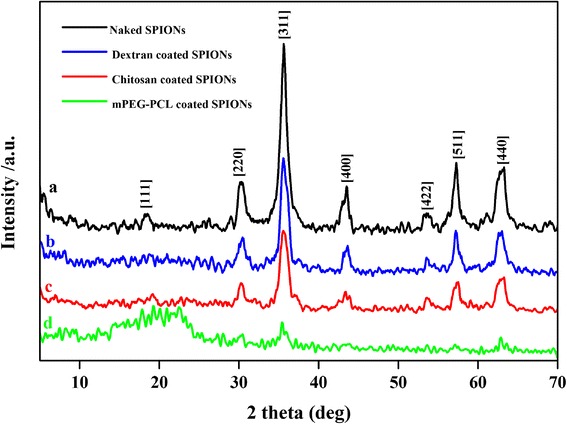
X-ray diffraction (XRD) patterns of naked SPIONs **a**, dextran coated SPIONs **b**, chitosan coated SPIONs **c** and mPEG-PCL coated SPIONs **d**

The crystalline properties of the naked and polymer coated SPIONs were analyzed by recording X-ray diffraction patterns (XRD). Figure 2 shows the X-ray diffraction patterns of the naked SPIONs and the dextran, chitosan and mPEG-PCL coated SPIONs. For the naked SPIONs the multiple peaks were observed at 2θ =18.25° (1 1 1), 30.06° (2 2 0), 35.63° (3 1 1), 43.48° (4 0 0), 53.78° (4 2 2), 57.33° (5 1 1) and 63.11° (4 4 0) which are indexed as those of inverse spinal structure of magnetite (JCPDS card No. 01-088-0866) (Fig. 2a). The XRD analysis is also indicative of the absence of the other types of iron oxides in synthesized product [22]. Of particular note was that for the chitosan and dextran coated SPIONs, the characteristic peaks did not disappear and still be seen, however the peak intensities of the diffraction peaks were weakened and width was broadened (Fig. 2b, c). Whereas in the case of mPEG-PCL coated SPIONs probably due to polymer amorphous properties and bilayer coverage (Fig. 2d), the characteristic peaks almost disappeared.

The average crystallite size was calculated using the Debye–Sherrer equation:

3 $$ D=\frac{K\lambda }{\beta \cos \theta } $$

Where *β* is the Full Width at Half Maximum (FWHM) of high intensity, *K* is Sherrer constant, *λ* is the X-ray wavelength and *θ* is the Bragg diffraction angle. The crystallite estimated size thus obtained from this formula were about 11, 13, 8 and 24 nm for the naked SPIONs, and the dextran, chitosan and mPEG-PCL coated SPIONs, respectively.

**Fig. 3:**
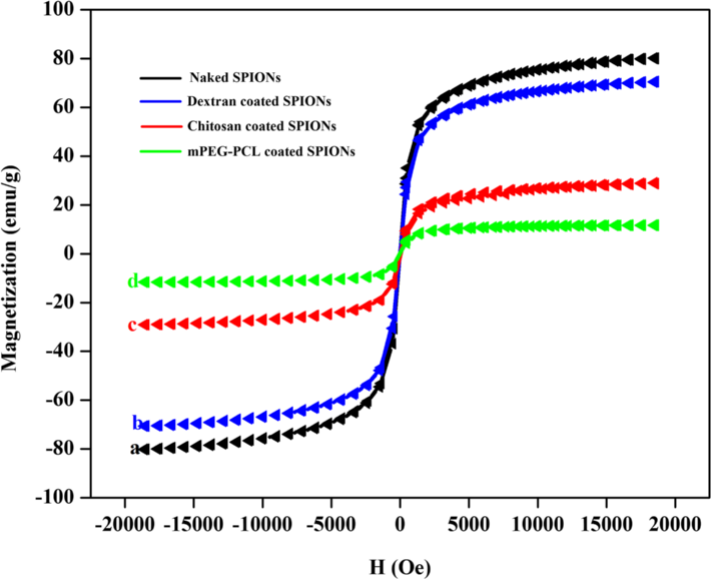
Magnetization curves of naked SPIONs **a**, dextran coated SPIONs **b**, chitosan coated SPIONs **c** and mPEG-PCL coated SPIONs **d** at room temperature

The magnetic properties of nanoparticles obtained via VSM technique. Figure 3 shows the hysteresis loops of the naked and various polymer coated SPIONs at room temperature. Due to fluctuation of magnetic moment by thermal energy, remanence and coercivity were about zero.

In the case of a ferrofluid the return of the magnetization to equilibrium state is determined by the sum of the Neel relaxation rate and the Brownian relaxation rate. It is believed that for a large particles, Brownian relaxation time is shorter than Neel relaxation time and subsequently, the viscous rotation determines the global relaxation. As it can be seen, for this case the magnetization curve is totally reversible because of the fast magnetic relaxation which causes the system to be remained at thermodynamic equilibrium [5]. As illustrated in the Fig. 3, all the samples exhibited a typical superparamagnetic behavior suggesting that SPIONs coated with polymeric shells can preserve their superparamagnetic properties. It could be found that the naked SPIONs presents the highest values of the magnetization (80.125 emu/g) while the saturation magnetization (M_s_) was found to be 70.572, 29.085 and 11.690 emu/g at 20000 Oe, for the dextran, chitosan and mPEG-PCL coated SPIONs, respectively. The results suggest that polymer coated SPIONs demonstrated a lower level of magnetization compared to that of the naked SPIONs. The observed trend of SPIONs magnetization correlates with the size of nanoparticles. It is known that for small magnetite nanoparticles because of large surface-to-volume ratio, the spin canting effect is not negligible thus magnetization decreases [18, 21]. The low magnetic susceptibility of mPEG-PCL coated SPIONs likely arises from a double coating of magnetite core by oleic acid as hydrophobic stabilizer and polymeric layer. However, this amount of saturation magnetization is sufficient for biological applications of ferrofluids as contrast agent. The magnetic properties of the naked and the polymer coated SPIONs is displayed in Table 1.

**Table 1:** The magnetic properties of naked SPIONs, dextran coated SPIONs, chitosan coated SPIONs and mPEG-PCL coated SPIONs

Preaperd NPs	Coercivity (Hci) G	Initial Slope emu/(gG)	Magnetization (Ms) emu/g	Negative (Hci) G	Positive (Hci) G	Retentivity (Mr) emu/g	Negative (Mr) emu/g	Positive (Mr) emu/g
Naked SPIONs	2.4059	0.023	80.152	−7.9299	−12.7420	0.16603	0.87937	0.54731
Dextran coated SPIONs	0.0850	0.021	70.572	−8.2975	−8.1267	0.00591	0.46993	0.48175
Chitosan coated SPIONs	12.5810	0.009	29.085	−31.4290	−6.2673	0.29041	0.14339	0.72421
mPEG-PCL coated SPIONs	1.2877	0.004	11.690	−0.7560	1.8194	0.01415	−0.02001	0.00830

**Fig. 4:**
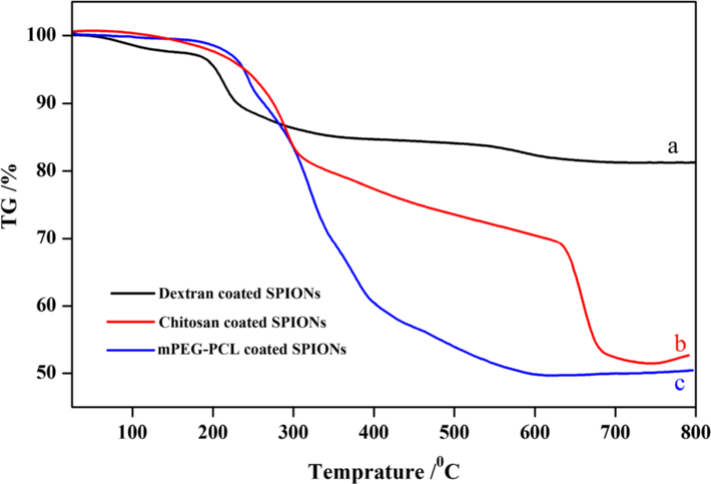
TGA curve of dextran coated SPIONs **a**, chitosan-coated SPIONs **b** and mPEG-PCL SPIONs **c**

In order to evaluate the extent of the polymer associated with the SPIONs, TGA analysis were accomplished under nitrogen atmosphere condition. As shown in Fig. 4, the TGA curves depict the changes of residual mass of the polymer coated SPIONs with temperature. As shown, for all curves a small weight loss of 2.42, 1.26, and 0.72 % for the dextran, chitosan, and mPEG-PCL coated SPIONs, respectively, within the first 150 °C which can be due to the loss of adsorbed water similar to that previously found in many systems based on polymer-coated SPIONs [23]. In the case of the dextran coated SPIONs, polymer decomposition is took place in two steps: 12.77 % between 150 and 380 °C presumably due to the breakdown of organic skeleton and 3.56 % at the range of 380–700 °C attributed to the complex degradation process. By considering these weight losses, the total amount of magnetite in sample is 81.25 %.

The TGA curve of the chitosan coated SPIONs also shows two distinct weight loss for polymer at 150–320 °C and 310–620 °C corresponding to 21.64 and 12.99 % of weight loss, respectively. The reports indicate that magnetite can be oxidized at elevated temperature up to 600 °C [24]. Thereby, the last weight loss in this thermogram can be ascribed to the magnetite oxidation which is occurred at the temperature of higher than 600 °C and subsequently indicating 42.56 % of iron oxide in the chitosan coated SPIONs. As shown in Fig. 4c, for the TGA curve of mPEG-PCL, the weight loss of 9.28 % at150–250 °C can be attributed to the evaporation of oleic acid and the weight loss of totally 33.53 % at 280–500 °C are as a result of copolymer decomposition. In this case also weight loss of 6.77 % can be related to the magnetite oxidation and consequently the residual weight of magnetite content was 50.43 %.

The average hydrodynamic diameter and size distribution of the naked and modified SPIONs were investigated by dynamic laser light scattering measurements at 25 °C in deionized water. Each measurement was repeated three times. The hydrodynamic sizes of the naked SPIONs (a), dextran coated SPIONs (b), chitosan coated SPIONs (c) and mPEG-PCL coated SPIONs (d) were 126.2 ± 9.179, 58 ± 10.594, 32.09 ± 6.766 and 42.23 ± 5.490 nm, respectively and the polydispersity indexes were 0.253 ± 0.008, 0.279 ± 0.009, 0.205 ± 0.004 and 0.264 ± 0.006, respectively. Large particle size of the naked SPIONs compared to that of polymer coated nanoparticles can be result of each nucleus surrounding by different hydrophilic polymers in the case of polymer coated SPIONs. This phenomenon ultimately forbids the addition growth of the nuclei.

The charge of the surface of nanoparticles was determined by zeta potential measurements. The naked SPIONs showed a negative zeta potential of −24.2 ± 0.494 mV. Following coating SPIONs with dextran the zeta potential increased to −16.9 ± 0.070 mV as a result of the interaction of the ions in aqueous dispersion with polysaccharide structure of dextran. The zeta potential of the chitosan coated SPIONs was measured to be +31.6 ± 0.919 mV. Such positive zeta potential is due to the presence of positively charged amino group of chitosan on the surface of SPIONs. mPEG-PCL coated SPIONs exhibited negative zeta potential of −21 ± 3.535 mV. Relatively high surface potential of all SPIONs could play a critical role in minimizing aggregation of particles and improvement of the colloidal stability of ferrofluid suspension.

**Fig. 5:**
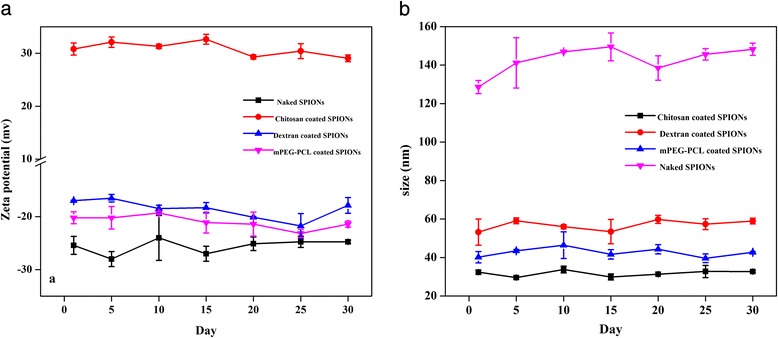
**a** Zeta potentials and **b** hydrodynamic diameters of different variants of the naked and coated SPIONs during a month. Each data point represents the mean ± S.D. (*n* = 3)

Stability of ferrofluids plays a critical role in biofate of nanoparticles. In order to evaluate the stability of SPIONs, zeta potential of different SPIONs were followed for one month. In fact, zeta potential of nanoparticles can severely affect the stability of ferrofluids. Figure 5 illustrates the changes of zeta potential and particle size of nanoparticles for one month. As it can be seen except the naked SPIONs that showed slight variation in the size and zeta potential, the polymer stabilized nanoparticles indicated no significant changes during this time. Since magnetic dipole interaction of polymers are zero or very small, so the presence of these polymers on the surface of nanoparticles results in fine colloidal stability of polymer coated SPIONs in aqueous media [18]. Therefore, polymer coatings on SPIONs can play a critical role in minimizing aggregation of particles, and improvement of the stability and prolonging circulation times.

**Fig. 6:**
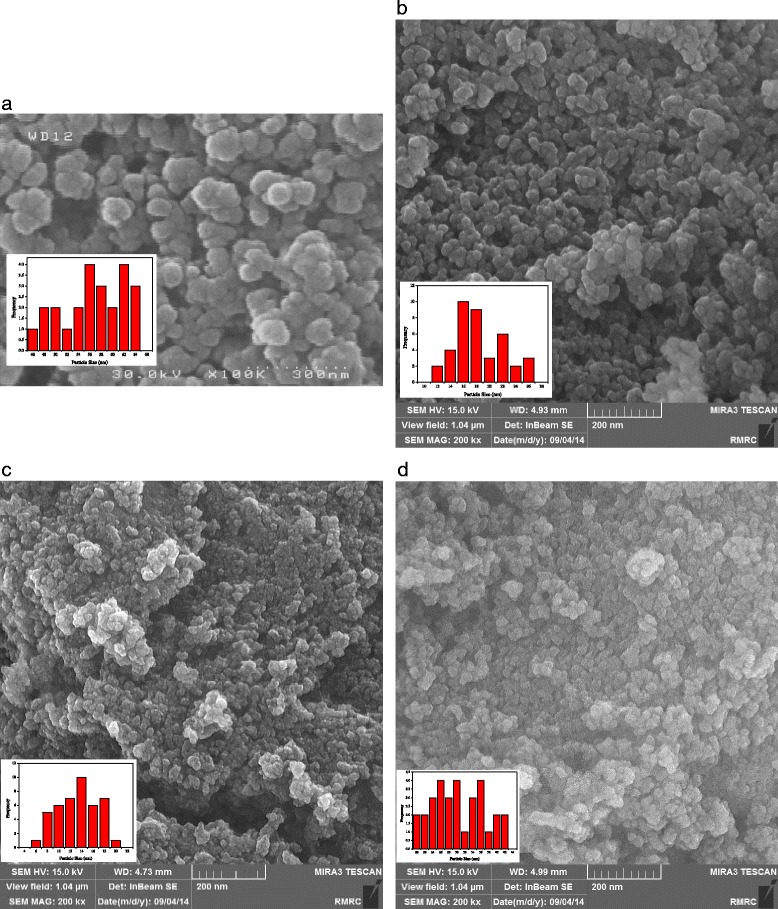
Field-Emission Scanning Electron Microscopy (FE-SEM) image of **a** naked SPIONs, **b** dextran coated SPIONs, **c** chitosan coated SPIONs and **d** mPEG-PCL coated SPIONs

The surface morphology and particle size of the naked SPIONs and polymer-coated SPIONs were observed by field-emission scanning electron microscopy (FE-SEM). FE-SEM images and corresponding histograms were shown in Fig. 6. The images reveal that most of the particles are quasi-spherical and SPIONs are apt to aggregate in the solid state since the surface energy is high. The related histogram of nanoparticles shows that the mean diameter of the naked SPIONs varied from 46 to 64 nm, while average particle size of coated particles does not approximately exceed 42 nm. By comparing the naked and polymer coated nanoparticles sizes, it can be concluded that the size of particles is significantly controlled by stabilizing agents. It is clear that the particle size obtained by DLS technique is much greater than those by FE-SEM which can be explained by the fact that in contrast to FE-SEM, DLS method measures the hydrodynamic diameter in suspension.

## MRI studies and relaxometric properties

SPIONs are commonly used as T_2_ MRI contrast agents and consequently they are able to decrease the MR signal intensity by dephasing of proton spins. Considering the biocompatibility of dextran, chitosan and mPEG-PCL, the effect of surface modification of SPIONs was investigated in terms of MR signal-enhancing property.

**Fig. 7:**
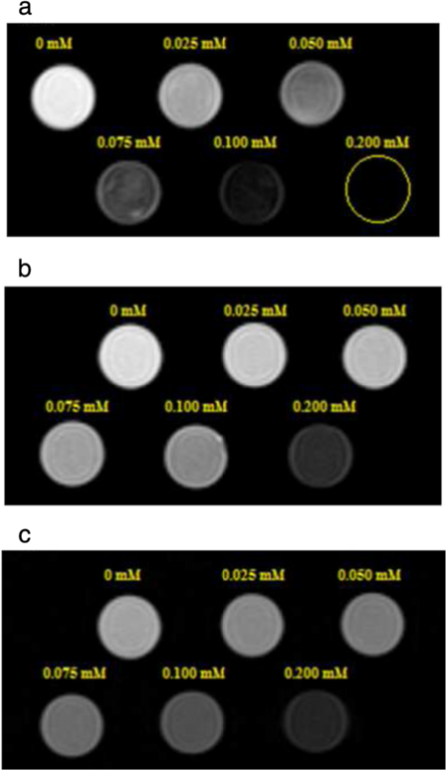
T_2_-weighted MRI images (1.5 T, spin-echo sequence: repitition time TR = 1600 ms, echo time TE = 108 ms) of the dextran coated SPIONs **a**, chitosan coated SPIONs **b** and mPEG-PCL coated SPIONs **c** at various iron concentration at 25 °C

The proton relaxivity measurements of the as-prepared polymer coated magnetite nanoparticles in aqueous solution with different Fe concentrations were performed to evaluate the feasibility of polymer coated magnetite nanoparticles as T_2_ MRI contrast agents. Figure 7 shows T_2_-weighted MR images of dextran coated SPIONs (a), chitosan coated SPIONs (b) and mPEG-PCL coated SPIONs (c) with iron concentrations of 0, 25, 50, 75, 100 and 200 μM in deionized water.

As shown in Fig. 7, The T_2_-weighted phantom images of polymer coated magnetite nanoparticles showed a significant negative dose dependent contrast enhancement which suggests them as an excellent T_2_ contrast agent under the T_2_-imaging sequences. From the results shown in Table 2, the images of the dextran coated SPIONs are darker than that of the chitosan and mPEG-PCL coated SPIONs at the same Fe concentration indicating corresponding high r_2_ relaxivity.

**Table 2:** The longitudinal relaxivity (r_1_, mM^−1^ s^−1^), transverse relaxivity (r_2_, mM^−1^ s^−1^), r_2_/r_1_values and R^2^ of polymer coated magnetite nanoparticles was calculated by plotting the T_1_ relaxation rate (1/T_1_) and T_2_ relaxationrate (1/T_2_) as a function of Fe concentration

Preaperd NPs	r_1_ (mM^−1^ s^−1^)	R^2^	r_2_ (mM^−1^ s^−1^)	R^2^	r_2_/r_1_
Dextran coated SPIONs	12.790	0.967	220.20	0.981	17.21
Chitosan coated SPIONs	4.708	0.799	91.44	0.943	19.42
mPEG-PCL coated SPIONs	4.174	0.990	86.46	0.990	20.713

The longitudinal relaxivity (r_1_, mM^−1^ s^−1^) and transverse relaxivity (r_2_, mM^−1^ s^−1^) of polymer coated magnetite nanoparticles was calculated according to the following equation:

4 $$ {R}_i=\frac{1}{T_i}={\left(\frac{1}{T_i}\right)}_0+{r}_iC $$

**Fig. 8:**
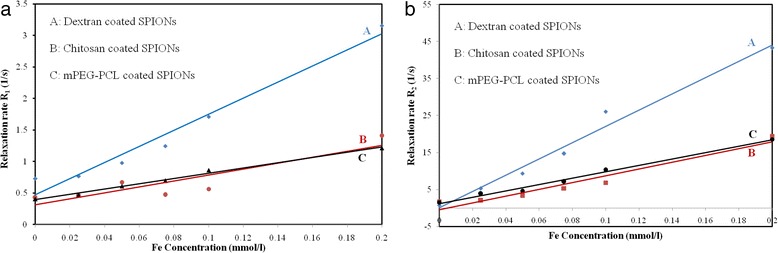
T_1_ relaxation rate plotted as a function of Fe concentration (mM) for polymer coated SPIONs **a**. T_2_ relaxation rate plotted as a function of Fe concentration (mM) for polymer coated SPIONs **b**

Where *R*_*i*_ is the relaxation rate, *T*_*i0*_ is the relaxation time in the pure water, *C* is the concentration of the contrast agent, and r_i_ is relaxivity [18]. By plotting the T_1_ relaxation rate (1/T_1_) and T_2_ relaxation rate (1/T_2_) as a function of Fe concentration a linear relationships were found for both of them (Fig. 8). The calculated r_1_, r_2_ and r_2_/r_1_ values for various polymer coated SPIONs are presented in Table 2.

The T_1_ relaxation rate (1/T_1_) as a function of Fe concentration of polymer coated SPIONs is presented in Fig. 8a. The findings reveal that for all three formulations, the longitudinal relaxation decreases as the iron concentration of the magnetic fluids increases. The slope of plots give the r_1_ values about 12.79, 4.708 and 4.171 mM^−1^ s^−1^ for the dextran, chitosan and mPEG-PCL coated SPIONs, respectively. Of particular note was the lower longitudinal relaxivity of mPEG-PCL coated SPIONs compared to that of dextran and chitosan coated SPIONs (Fig. 8a). It is clear that the relaxivities greatly is affect by distance of the aqueous medium from the magnetite core. On the other hand, the hydrophobicity/hydrophilicity of the coatings has an impact on the diffusion of water within polymeric layer. Thereby, it can be postulated that the presence of hydrophobic inner shells of mPEG-PCL coated SPIONs including oleic acid and PCL layers will exclude water molecules and consequently extend the distance of water molecules from the magnetite core and finally result in low longitudinal relaxivity [25].

R_2_ relaxivity of magnetite nanoparticles is given by the following formula:

5 $$ {R}_2=\frac{1}{T_2}=\frac{\left(\frac{256{\pi}^2{\gamma}^2}{405}\right){V}^{\ast }{M}_s^2{a}^2}{D\left(1+L/a\right)} $$

Where *a* is magnetite core radius, *M*_*s*_ is the saturation magnetization nanoparticles, V* is the volume fraction of magnetite core and *L* is the thickness of an inscrutable surface coating. According to equation 5, the R_2_ relaxivity decreases once coating layer thickness increases. On the other hand, surface coating affect the movement of water molecules [18]. The specific relaxivity (r_2_) of the dextran coated SPIONs was calculated to be 220.20 mM^−1^ s^−1^, which was significantly higher than that of the chitosan coated SPIONs (91.44 mM^−1^ s^−1^) and mPEG-PCL coated SPIONs (86.46 mM^−1^ s^−1^) (Fig. 8b). The remarkable r_2_ relaxivities of dextran coated SPIONs compared to that of chitosan and mPEG-PCL coated SPIONs can be explained by its high saturation magnetizations, high crystallinity and larger hydrodynamic diameter as well as dextran hydrophilicity [5, 18]. In fact, high hydrophilicity of dextran lead to strong hydrogen bond between polymer and water molecules and prevent water molecules diffusion from the nanoparticles surface toward magnetic core which in turn can be considered as a reason for its high r_2_ relaxivities. Further experimental results supporting above point can be seen elsewhere [18]. According to the DLS analysis, it can be seen that the highest r_2_ relaxivities of formulations is belonged to the dextran coated SPIONs which is the largest particle according to the DLS analysis. This trend is in accordance with the literature data [5, 18]. The high r_2_ relaxivities of dextran coated SPIONs can be also attributed, in part, to its high crystallinity as evidenced by the XRD data similar to that previously found [18]. The most important feature of the single monodomain is its anisotropy energy that is given by the following equation:

6 $$ {E}_a={K}_aV $$

Where *V* is the crystal volume and *K*_*a*_ is the anisotropy constant. Clearly, the anisotropy energy increases by increasing the crystal radius, and subsequently the Neel relaxation time is influenced by the anisotropy energy [5]. It is important to note that in addition to magnetic properties of the core, hydrodynamic diameter, and crystallinity, several parameters such as composition, doping, assembly of magnetite-based nanoparticles also strongly affect the R_2_ relaxivity of magnetite nanoparticles [18]. Analogously with the longitudinal relaxation results, the mPEG-PCL coated SPIONs exhibit lower r_2_ values than the dextran and chitosan coated SPIONs.

Typically contrast agents with r_2_/r_1_ ratio of larger than 2 and up to 40 are considered as T_2_ contrast agents, while for T_1_ contrast agents, this ratio is relatively low [26]. The r_2_/r_1_ values of the prepared polymer coated magnetite nanoparticles were around 20 which is higher than that of Resovist, commercially available MRI contrast agent [9]. From the discussion above, it can be concluded that all as prepared formulations are feasible to be used as negative MRI contrast agents. However, in view of the r_2_/r_1_ value of different formulations, it can be concluded that mPEG-PCL coated SPIONs compared to the chitosan and dextran coated SPIONs due to higher r_2_/r_1_ value, can be considered as promising candidate as T_2_ contrast agent. The reason behind these results, presumably lies on presence of the hydrophobic layer on the surface of mPEG-PCL coated SPIONs. This study has hinted at the potential of mPEG-PCL coated SPIONs as T_2_ contrast agent but also illustrate the need for more trials and more work.

## Conclusion

Synthesis of the core − shell nanostructures composed of magnetite (Fe_3_O_4_) nanoparticles stabilized with various polymer coatings such as dextran, chitosan and mPEG-PCL via a simple coprecipitation method was achieved. All the as-prepared polymer coated SPIONs had excellent water dispersion and colloidal stability. FT-IR and TGA confirmed that polymer chains had been effectively coated on the surface of SPIONs. Field-emission scanning electron microscopy (FE-SEM) confirmed the formation of quasi spherical nanostructures with the average particle size about 50 nm. The VSM analysis showed that different polymer coated magnetite nanoparticles are superparamagnetic with high saturation magnetizations value (M_s_) of about 70.572, 29.085 and 11.690 emu/g at 20000 Oe for the dextran, chitosan and mPEG-PCL coated SPIONs, respectively, that is sufficient for their application as MRI contrast agents. X-ray diffraction (XRD) analysis proved highly crystalline magnetite particles with an inverse spinel structure. All SPIONs exhibit high r_2_ relaxivities about 220.20 mM^−1^ s^−1^, 91.44 mM^−1^ s^−1^ and 86.46 mM^−1^ s^−1^ for the dextran, chitosan and mPEG-PCL coated SPIONs, respectively. The value of r_2_/r_1_ ratios of prepared SPIONs is higher than that of some commercial contrast agents such as Resovist. The results of this study have indicated the possibility of using polymer stabilized SPIONs especially mPEG-PCL coated SPIONs as potential T_2_ MRI contrast agents.
